# Tamoxifen from Failed Contraceptive Pill to Best-Selling Breast Cancer Medicine: A Case-Study in Pharmaceutical Innovation

**DOI:** 10.3389/fphar.2017.00620

**Published:** 2017-09-12

**Authors:** Viviane M. Quirke

**Affiliations:** School of History, Philosophy and Culture, Faculty of Humanities and Social Sciences, Oxford Brookes University Oxford, United Kingdom

**Keywords:** synthetic anti-estrogen, contraceptive pill, breast cancer, chemoprevention, risk evaluation and management, adjuvant therapy

## Abstract

Today, tamoxifen is one of the world's best-selling hormonal breast cancer drugs. However, it was not always so. Compound ICI 46,474 (as it was first known) was synthesized in 1962 within a project to develop a contraceptive pill in the pharmaceutical laboratories of ICI (now part of AstraZeneca). Although designed to act as an anti-estrogen, the compound stimulated, rather than suppressed ovulation in women. This, and the fact that it could not be patented in the USA, its largest potential market, meant that ICI nearly stopped the project. It was saved partly because the team's leader, Arthur Walpole, threatened to resign, and pressed on with another project: to develop tamoxifen as a treatment for breast cancer. Even then, its market appeared small, because at first it was mainly used as a palliative treatment for advanced breast cancer. An important turning point in tamoxifen's journey from orphan drug to best-selling medicine occurred in the 1980s, when clinical trials showed that it was also useful as an adjuvant to surgery and chemotherapy in the early stages of the disease. Later, trials demonstrated that it could prevent its occurrence or re-occurrence in women at high risk of breast cancer. Thus, it became the first preventive for any cancer, helping to establish the broader principles of chemoprevention, and extending the market for tamoxifen and similar drugs further still. Using tamoxifen as a case study, this paper discusses the limits of the rational approach to drug design, the role of human actors, and the series of feedback loops between bench and bedside that underpins pharmaceutical innovation. The paper also highlights the complex evaluation and management of risk that are involved in all therapies, but more especially perhaps in life-threatening and emotion-laden diseases like cancer.

## Introduction

Today, tamoxifen (brand name Nolvadex) is one of the world's best-selling hormonal breast cancer drugs. However, it was not always so. Compound ICI 46,474 (as it was first known) was synthesized in 1962, quite unusually for the time, by a female chemist: Dora Richardson, who was responsible for making triphenylethylene derivatives within a project to develop a contraceptive pill in the pharmaceutical laboratories of the British chemical group ICI (now part of AstraZeneca). Although designed to act as an anti-estrogen, the compound was found to stimulate, rather than suppress ovulation in women. This, and the fact that at first it could not be patented in the USA, its largest potential market, meant that ICI nearly stopped the project. If it was saved, it was partly because the team's leader, Arthur Walpole, threatened to resign, and pressed on with another project: to develop tamoxifen as a treatment for breast cancer. Even then, its market appeared small, because at first it was mainly used as a palliative treatment for advanced breast cancer. An important turning point in tamoxifen's journey from orphan drug to best-selling medicine occurred in the 1980s, when the results of clinical trials showed that it was also useful as an adjuvant to other forms of therapy in the early stages of the disease. Later, trials demonstrated that it could prevent its occurrence or re-occurrence in women at high risk of developing breast cancer. Thus, it became the first chemopreventative for any cancer, helping to establish the broader principles of chemoprevention, and extending the market for tamoxifen and similar drugs further still.

Hailed as a pioneering medicine that has saved the lives of thousands of women^1^, much has been written about tamoxifen, especially in recent years by Craig Jordan, the researcher who was influential in the latter part of its history (Maximov et al., 2016). However, as the public's and the medical profession's dependence on drugs not only to treat, but also to prevent an ever growing variety of conditions has come under increasing scrutiny (Greene, 2007), tamoxifen has also been investigated by sociologists as an example of what they describe as the “biomedicalization” of society, i.e., the shift in the use and meaning of drugs from treatment to prevention, involving a cost-benefit calculation that is seldom openly discussed (Fosket, 2010, pp. 341–348; Löwy, 2010, pp. 185–188; Löwy, 2012).

There is another strand in the literature, which is somewhat less well-developed, and concerns tamoxifen at once as an emblematic and an idiosyncratic example of pharmaceutical innovation. For the history of tamoxifen suggests a model of pharmaceutical innovation that is far more complex than a linear model from bench to bedside (Schwartzman, 1976; Howells and Neary, 1988; Gambardella, 1995; Landau et al., 1999). Rather, it incorporates numerous dead ends, feedback loops, as well as serendipitous observations made by individual researchers (and associated with other discoveries, in this instance the isolation of the estrogen receptor). Hence, the scientists whose work has shaped pharmaceutical innovation are an important part of the story—in the case of tamoxifen, not only Richardson, but also Walpole, the biologist who led the research team at ICI and provided the link between the different projects within which tamoxifen was developed (Jordan, 1988), and, for the later stage in the drug's tortuous journey, Jordan.

At a time when the drying up of old drug pipelines has led to anxieties about the end of the Therapeutic Revolution and the need to find new models of drug discovery to replace those which produced many of the blockbuster drugs we know today, tamoxifen therefore presents an opportunity to explore the historically contingent nature of pharmaceutical innovation, addressing several of the questions posed by the editors (see their introduction to this special issue). Using the research and development reports of the company that developed the drug (ICI)^2^, an unpublished history of tamoxifen, written by Richardson and accompanied by letters from patients^3^, as well as some of the numerous publications on the topic, the paper will show how the early history of the drug shaped its fate in the medical marketplace, and therefore deserves to be better understood than it is at present. The paper argues that its origins as a contraceptive pill rather than a cancer remedy meant that concerns over side-effects, alongside its ability to counteract the action of estrogen, dominated the company's research and development agenda. Hence patients' voices, which provided indications the drug's safety and efficacy at once directly and indirectly, helped to define this agenda, and the absence of side-effects relative to its anti-estrogenic activity would become one of the key selling points of tamoxifen as an anti-cancer drug compared to alternative treatments.

However, because of its very ability to prolong life in women suffering from breast cancer, tamoxifen was later found to have a number of potentially serious long-term side-effects, which range from pulmonary thrombosis to endometrial cancer. Nevertheless, its usefulness in treating and preventing a major cause of death in women has meant that, to this day, it remains on the WHO's List of Essential Medicines (WHO, 2015). This paper will therefore also highlight the complex evaluation of risk that is involved in all therapies, but more especially perhaps in diseases as threatening and emotionally charged as cancer, not only at the regulatory and clinical levels, but also at the individual level of the patient.

## Results

Before focusing on the development of tamoxifen, it is useful to describe the background for the different projects that led first to its synthesis, second to its early trajectory as an anticancer drug, for it illustrates not only the non-linear nature of pharmaceutical innovation, but also the lengthy accumulation of in-house scientific knowledge and technical know-how which underpins it, and yet is rarely brought to the fore in histories of drug discovery (Weatherall, 1990; Sneader, 2005; Ravina, 2011).

### The use of sex hormones and synthetic analogues in cancer

The link between hormones and cancer has been known at least since 1916 (Lathrob and Loeb, 1916). However, their usage in the treatment of cancer depended on their isolation, purification and chemical determination, which was not achieved until the 1930s in the case of sex hormones. One such hormone was the follicular hormone (Follicle-Stimulating Hormone, FSH), which was prepared by the Roussel Laboratories, a French company specializing in biologicals, and supplied to Antoine Lacassagne at the Institut du Radium in Paris. Using this hormone, Lacassagne was able to show a direct link between estrogens and the appearance of breast cancer in mice (Lacassagne, 1932, 1936). But natural estrogens were difficult to obtain in the quantities required for large-scale experiments. A major turning point occurred when E.C. (later Sir Charles) Dodds, working at the Middlesex Hospital in London in collaboration with researchers at the Dyson Perrins Laboratory in Oxford, discovered that the synthetic compound stilboestrol had estrogenic properties (Dodds et al., 1938). Dorothy Crowfoot (later known by her married name, Hodgkin), who worked nearby at Oxford University's Inorganic Chemistry Department, established using X-ray crysallography that its chemical structure resembled estrogen (Carlisle and Crowfoot, 1941). Inexpensive to make and apparently well tolerated in patients, stilboestrol was therefore widely prescribed for cases of estrogen deficiency, especially in menopausal women (Sneader, 2005, p. 197). Although it would later be linked to cases of vaginal or cervical adenosarcomas in daughters of women who had been prescribed the drug in their first trimester of pregnancy (to avoid unwanted abortion; see Gaudillière, 2014), it was also the first synthetic drug to be used for treating cancer (Weatherall, 1990, pp. 217–218). Indeed, in 1939, Charles Huggins of the University of Chicago successfully treated cases of prostate cancer with stilboestrol (known as diethylstilbestrol in the US; Huggins and Hodges, 1941), and by 1950 a co-operative trial had shown that the synthetic estrogen was effective in delaying the progress of this type of malignant disease (Nesbitt and Baum, 1950). However, breast cancer proved more difficult to treat, as it could either be inhibited or stimulated by administration of estrogen.

Following the publication of Dodds' findings, synthetic substances with a similar structure were examined for estrogenic activity, such as, triphenylethylene. These substances, which could not only be mass produced, but also be chemically modified to obtain derivatives with *anti*-estrogenic activity, therefore became compounds of choice for studies in Britain and elsewhere. One of the organizations that studied it was ICI, which I turn to now.

### ICI and cancer research

The company's interest in cancer was a long-standing one. When triphenylethylene was found by Charles Scott, a researcher in Edinburgh, not only to be active by mouth, like stilboestrol, but to have a durable estrogenic action, and therefore to have potential as an alternative to stilboestrol, Arthur Walpole, a biologist who had joined ICI's Medicinal Section in 1938, began carrying out some exploratory work with the substance. This work led to the synthesis of various triphenylethylene derivatives, including triphenylmethylethylene (M 612) and triphenylchloroethylene (registered in 1940 under the name Gynosone)^4^. In 1942, these compounds were supplied by the company for trials in breast cancer to Alexander Haddow of the Chester Beatty Institute in London, Edith Patterson at the Christie Hospital in Manchester and their collaborators. Although improvements were only temporary, there was clear evidence that Gynosone in particular caused regression and therefore could be beneficial in the treatment of breast cancer (Haddow et al., 1944; Walpole and Paterson, 1949).

Meanwhile, on the other side of the Atlantic, the compounds known as “nitrogen mustards,” which were being studied as part of a chemical warfare research programme, were shown to inhibit the growth of blood and lymph tumors by Goodman and Gilman at the University of Yale, a discovery often hailed as the beginning of cancer chemotherapy^5^. Despite this wartime work being top-secret, Walpole and Haddow were also able to investigate these compounds, thanks to an Anglo-American agreement to exchange scientific information (Weatherall, 1990, p. 218). Another, parallel study relating to cancer at ICI involved anti-metabolites. Following the discovery that ICI's novel anti-malarial drug Paludrine was converted in the body to cycloguanine, an active metabolite which interferes with purine biosynthesis, and spurred by the announcement that Burroughs Wellcome's drug 6-MP was effective against leukemia, the search for anti-metabolites began at ICI under the leadership of Frank Rose, who had run their anti-malarial programme during the war. Rose became Research Manager of the Chemistry Department in 1954, whilst remaining involved in bench work. As well as the search for alkylating agents, synthetic estrogens, and anti-metabolites, Rose also encouraged investigations into carcinogenesis, which was a rare interest for researchers working on cancer chemotherapy at that time (Suckling and Langley, 1990, pp. 507–508).

At first, ICI's approach to cancer was therefore largely empirical, involving the synthesis of derivatives of compounds that had known anti-tumor properties, without a formal cancer research programme. However, once plans had been made to build a pharmaceutical research center at Alderley Park near Manchester, and ICI started organizing its research in team projects, Cancer became such a project in 1955. The project was entitled “Cancer and Viruses: antibacterials^6^,” and its team leader was the biologist E. Weston Hurst. Alderley Park opened in 1957, and between 1957 and 1960 Cancer and Viruses separated into two different projects.

During that time Cancer was merged with a new project to find an oral contraceptive, led by Arthur Walpole. Then, in 1960, the discovery of the natural antiviral substance interferon, and ICI's involvement in its study in collaboration with the Medical Research Council (see Pieters, 2005, Chapters 5–6), led to Viruses and Cancer coming together again. Oral Contraception therefore split away from Cancer, with Walpole working in parallel on both projects. His involvement in the Oral Contraception project (which in 1963 was re-named “Endocrinology,” and later “Fertility,” reflecting a gradual change in the research emphasis) would ensure that breast cancer remained an important focus for both his teams. It was within this Oral Contraception project that tamoxifen (Nolvadex), a triphenylethylene derivative, was synthesized and subsequently developed, initially as a contraceptive pill.

### ICI, oral contraception, and the origins of tamoxifen

The first contraceptive pill had been synthesized in the early 1950s, and in 1956 Walpole wrote a survey entitled “The technical possibility of oral contraception^7^,” which—as had become customary within ICI by that time (see Quirke, 2005)—gave an overview of the field to enable ICI to decide whether or not it was worth entering.

Walpole began by introducing the context in which such a pill would be developed. In doing so, he showed the extent to which contemporary concerns, which included anxiety over population growth, a decrease in death rates, food shortages, and an awareness of important differences between the developed and developing world, were internalized and acted upon by companies such as ICI. Then, in the main body of his report, Walpole enumerated the requirements for contraception:

1. it should not “offend social or religious scruples” and as little as possible the “aesthetic feelings” of those who might wish to avail themselves of it (however, he added, such considerations remained outside the scope of experimental biology);
2. it should be cheap enough to be readily available and simple enough to use by any people “intelligent enough to realize the possible consequence of coitus and to know whether or not they wished to conceive”;
3. it should be effective over a known period of time with no prejudice to subsequent fertility;
4. it should not depend on a local action contemporaneous with coitus or any form of treatment which must be timed in a complex or critical manner in relation to the menstrual cycle;
5. it should involve only occasional dosage by mouth.

After describing what at the time was understood about the physiology of reproduction, he went on to list the technical possibilities of contraception at different stages in the reproductive cycle, from (a) spermatogenesis and sperm, to (b) ovulation and ovum, (c) fertilization, (d) fertilized ovum, (e) implantation of embryo, and lastly (f) development of embryo.

On the basis of substances already known to act as contraceptives, he concluded that it “would seem possible to produce temporary infertility in men by giving androgens, and of these methylsterone is active by mouth.” He added that it was also possible “either to prevent conception or interrupt pregnancy at a very early stage in women by giving estrogens by mouth,” but that such treatments must be free from undesirable side-effects. Among the newer partly synthesized steroids now becoming available, he believed that substances might be found that were be more specifically antagonistic toward progesterone (anti-progestins), and he argued that these would seem more suitable for continued use^8^. Other substances from natural sources, such as, the *Lithospermum ruderale*, a North American plant with a small white flower that could also be found in English hedgerows and was being investigated at the time by the Medical Research Council (Marks, 2001, pp. 49–50), appeared to him as “rather more suspect,” and he acknowledged that clinical evidence was lacking, not only concerning these natural compounds, but also human contraception more generally.

As to the other substances that might be considered for contraception, toxicity was a major problem, such as the anti-folic drug aminopterin, for not only did it act as an early abortifacient, but it carried serious toxic hazards, like some of the other anti-metabolites. Similar concerns were associated with biological alkylating agents, which were potentially mutagenic and carcinogenic. Hence, taking into account both the requirements for contraception and the need to avoid toxic effects, especially since contraceptive substances were intended for use in normally young and healthy adults (Oudshoorn, 2002, pp. 123–157), the search for triphenylethylene derivatives, alongside investigations of natural and part-synthesized steroids, became the preferred course of action, as evidenced by ICI's research reports^9^.

ICI were not alone in pursuing the triphenylethylene route. Indeed, when Leonard Lerner, a researcher working on a cardiovascular research program at the American drug company Merrell, reported in 1958 that a newly synthesized compound, MER 25 (ethamoxytriphetol), not only resembled structurally triphenylethylene, but had anti-estrogenic activity on both spayed and intact female rats, his discovery stimulated laboratory research and clinical investigation of other potential anti-fertility agents among triphenylethylene derivatives. ICI considered acquiring the drug under license from Merrell in order to study and potentially exploit it as a contraceptive, but interest in it waned, for in the meantime ICI had found that another compound, ICI 22,365 [N:N-bis (allylthiocarbamyl) hydrazine], which they employed in analytical chemistry and were currently investigating as an anti-parasitic for use in the poultry industry, prevented the development of sex organs and secondary characteristics such as the emergence of combs in chicks^10^. This finding led Walpole's team, which at that stage included G. E. Paget and J. K. Walley working on the biological side (while Dora Richardson and G. A. Snow worked on the chemistry), to test the compound in male and female rats, producing evidence that it caused a selective and reversible inhibition of the gonadotrophic functions of the pituitary in rats, and prevented pregnancy either by inhibiting ovulation, or by preventing implantation (the precise mechanism of action was yet unclear). In a report written in September 1960, Walpole wrote that the compound not only provided an interesting lead in oral contraception, but also in hormone-dependent cancers of the prostate and breast, and it was decided that “if an alternative patentable compound were found which, in laboratory tests, proved superior (or even equivalent to it), then this compound should replace 22,365 in clinical studies^11^.”

The most promising compound to come out of this programme, ICI 33,828 (which had a similar structure to 22,365), was therefore tested in pre-menopausal patients with mammary carcinoma, which was justified on the grounds that it might have a therapeutic as well as an anti-fertility effect. It was also tried in prostatic cancer, however the clinicians involved in these trials at the MRC Clinical Endocrinology Unit in Edinburgh received complaints from patients about nausea, anorexia, and occasional vomiting. Walpole also discovered that, before trials with 33,828 could begin, 22,365 had been given in November 1960 to a psychotic patient who was 15 weeks pregnant in order to induce abortion. However, the drug had failed to terminate the pregnancy, and estrogen excretion had remained unaffected by the treatment. The fetus, which had therefore had to be removed surgically, appeared normal. At the same time as plans for more extensive clinical studies, preferably closer to home so that his team could be more directly involved in the trials, Walpole therefore also made plans to develop more sensitive assay methods for gonatrophins in urine, blood, and pituitary, to better assess the clinical effects of their lead compound, and obtain more reliable measures of activity in animal experiments^12^. Shortly afterwards, in 1962, Mike Harper, a young endocrinologist who would play a significant part in the tamoxifen story, was invited to join the team.

Meanwhile, at Merrell, researchers had pressed on with the search for novel triphenylethylenes and in 1961 discovered that MRL 41 (also known as clomiphene, or chloramiphene, brand name Clomid), which was in fact an ether derivative of Gynosone, also inhibited pituitary gonadotrophins although it showed weak estrogenic activity. Remembering the earlier trials with Gynosone and M 612, Walpole therefore suggested to his team that they develop and examine an ether derivative of M 612^13^. The compounds they prepared in 1961 not only inhibited implantation of the fertilized ovum in the rat at a low dose (below that at which they would show estrogenic activity), but with the addition of a methoxy group they also had a greater duration of action. After the arrival of Harper, whose new series of biological tests helped to produce a clearer picture of the structure–activity relationships of triphenylethylenes, the programme of chemical synthesis was therefore stepped up. The team had grown, and as well as Walpole, Walley, and Richardson, it now included several members of the Biology Group: A. M. Barrett, M. J. K. Harper, G. E. Paget, Miss J. M. Peters, and J. M. Thorp, of the Chemistry Group: R. Clarkson, E. R. H. Jones, J. K. Landquist, B. W. Langley, W. S. Waring, and of the Biochemistry Group: W. A. M. Duncan. It was hoped that, with such increased resources, ICI could improve upon both clomiphene and a new Upjohn product with similar activity, U 11,555, by finding alternatives with less estrogenic and pituitary-inhibitory activity relative to their anti-fertility activity. For, by then, clinical studies of ICI 33,828 had produced disappointing results: not only did it have unpleasant and worrying side effects (nausea, drowsiness, a fall in thyroid function measured by thyroidal I^132^ uptake, and a rise in serum cholesterol)^14^, but the inhibition of ovulation could not be achieved without suppressing menstruation, which made it undesirable as an oral contraceptive in women^15^.

Among the newly synthesized triphenylethylenes, Harper drew up a short list for further study, primarily as potential anti-fertility agents. These included the dimethylamino ethoxy compound ICI 46,474 (later known as tamoxifen, brand name Nolvadex). It had been synthesized in 1962 by Richardson, and Harper selected it for additional tests and for preliminary toxicity studies. At the same time, the company lodged patent applications to protect ICI 46,474 and related compounds from competitors^16^. As well as providing basic data on these compounds, Patent GB1013907 covered a number of potential therapeutic uses, including cancer. It read:

The alkene derivatives of the invention are useful for the modification of the endocrine status in man and animals and they may be useful for the control of hormone-dependent tumors or for the management of the sexual cycle and aberrations thereof. They will also have useful hypocholesteraemic activity^17^.

### ICI 46,474 (1962–67)

Although marred by a number of dead ends, which were partly due to ICI's strategy of closely following their competitor's activities and using their compounds as leads in the search for new, patentable products, the early phase of the Oral Contraception programme shaped tamoxifen and determined its future in many ways. The compounds developed within this programme were designed to act as contraceptive pills, yet from the beginning their usefulness in breast cancer was explored in close parallel. This dual objective was pursued as a result of Walpole's own research interests, and thanks to the fruitful collaborations he established both with endocrinologists and with clinicians working in cancer. The feedback loops between bench and bedside which this relationship created, and which led to a series of twists and turns that would become the hallmark of the tamoxifen story, meant that the compounds functioned both as research tools to study hormone function and metabolism in the laboratory, and as experimental treatments in the clinic. Importantly, the dual objective of developing a contraceptive pill whilst assessing the usefulness of compounds in breast cancer (even if as we have seen this was also a means of testing drugs before administering them to healthy women), also meant a constant preoccupation with side effects, and the low toxicity of tamoxifen relative to its potency would turn out to be one of its crucial advantages over its competitors.

A triphenylethylene derivative, with groups and side chains to enhance its anti-estrogenic and pituitary-inhibitory effect and prolong its duration of action, without interfering with its anti-fertility activity, ICI 46,474 had been demonstrated as the most potent and least toxic of all the compounds tested by June 1964^18^. But what exactly was it? In the process of gathering data for patent applications, scaling up production and preparing a submission to the newly formed Committee on Safety of Medicines (CSD), uncertainty arose as to the precise structure of the compound. Using an NMR spectrometer recently acquired by the company, in 1964 G. R. Bedford, a spectroscopist who had joined ICI's Pharmaceutical Division in 1963, showed that many of the active compounds synthesized so far were a mixture of isomers. However, it was unclear in which isomer the anti-estrogenic activity resided (did it reside in the *cis*, or the *trans* isomer?). The isomers were separated by fractional crystallization by Richardson. This represented quite a feat at the time^19^, and revealed ICI 46,474 to be more active as an anti-implantation agent than its *cis* isomer ICI 47,699, which was more estrogenic (Bedford and Richardson, 1966; Harper and Walpole, 1966). In the meantime, Merrell had carried out a spectroscopic analysis of their own drug clomiphene, and disagreed with ICI's interpretation of the spectroscopic data, attributing the anti-estrogenic activity to the *cis*, not the *trans* isomer. The controversy led to some confusion among researchers, and eventually the matter was settled by X-ray analysis, which confirmed ICI's findings that the anti-estrogenic activity did indeed reside in ICI 46,474, that is to say in the *trans* isomer of the compound (Kilbourn et al., 1968).

So how did tamoxifen work? Before making a submission to the CSD, which in the wake of the thalidomide disaster had been set up to review all laboratory data on potential drugs in advance of their introduction into human patients, a basic understanding of their mechanism of action, as well as knowledge about any toxic effects, had to be achieved (see Quirke, 2012a). Therefore, unsurprisingly perhaps since it was intended for use in contraception, the first teratogenic test ever to be performed by ICI was carried out with tamoxifen. At the very low doses necessary to allow implantation of the fertilized ovum, rat offspring developed a deformity called “kinky ribs.” However no such effects could be seen in rabbits or in primates, and it was later concluded that since ICI 46,474 restricts uterine growth, the deformity was caused by mechanical contraction and therefore could not considered a true teratogenic effect^20^.

Tamoxifen was most effective in preventing implantation in rats when given on day 4 of the pregnancy, and virtually inactive on day 5. This suggested that it acted by interfering with a crucial event that had already occurred by the 5th day. It was suspected that ICI 46,474 prevented implantation by interfering with the critical estrogen release on the uterus that occurs between 12 and 20–21 h on the 4th day^21^. However, it was unclear whether the estrogen released at this time acted directly on the uterus or whether its action was mediated by vasodilating amines such as histamine. As there was evidence to support the latter hypothesis, ICI 46,474 was thought to act either as a direct estrogen antagonist, or by preventing the release of histamine, or as an antagonist of the amine. To explore this hypothesis, whilst carrying out further toxicity tests, experiments were devised in additional animal species (as well as rats, in mice, rabbits, dogs, monkeys, and sheep, for by then the compound was also being considered for use in veterinary medicine)^22^. These experiments revealed considerable species specificity, and by 1965 doubts had arisen whether an “estrogen surge” was necessary for ovo-implantation in humans, as it was in rats, and whether at the dosage required to oppose estrogen sufficiently to inhibit implantation ICI 46,474 would cause menstrual irregularities, therefore whether the compound would prove effective and be acceptable as an oral contraceptive^23^. Although it was still hoped that ICI 46.474 would provide a welcome alternative to the now familiar method of using mixtures of orally active estrogens and gestagens (also known as progestogens) to inhibit ovulation while at the same time producing withdrawal bleeding to replace spontaneous menstruation, a method which was considered too costly and too complicated for use in underdeveloped communities, it was felt that such doubts could only be “settled in the clinic^24^.” However, first, the team needed to ascertain whether or not ICI 46,474 would produce irreversible damage to the ovaries or uterus, and for this studies in monkeys, particularly pig-tail monkeys in which changes in the reproductive cycle were found to most closely resemble those in man^25^, were deemed to be the most helpful.

### The first collaborative trials (1967–71)

While these further studies were being carried out, ICI began planning a trial with Dr. Klopper at Aberdeen, for the induction of ovulation in amenorrheic women rather than contraception^26^. Indeed, by then, clomiphene had been found to stimulate ovulation and prolong luteal function in amenorrheic women, and in 1967 was approved for the treatment of infertility in the US^27^. Moreover, obtaining approval to evaluate ICI 46,474 in oral contraception was problematic, not only because it involved long-term administration, but because of persisting fears among British gynecologists that it might lead to fetal malformation. In their eyes, unlike the conventional pill which contained familiar ingredients such as estrogens and progestins that had traditionally been given to pregnant women without harm to the fetus, evidence of a lack of teratogenic effect in animal experiments with an unknown compound like ICI 46,474 did not constitute an adequate safeguard. Therefore, they believed that the first women to receive ICI 46,474 as a contraceptive must be offered an abortion, but under the terms of the 1967 Abortion Act this could only be offered to a very limited number of women^28^. Two solutions to this conundrum were envisaged: (1) to arrange a consortium of gynecologists to contribute such patients to a central unit in the hope of collecting a reasonable number fairly quickly; (2) to go abroad to a country, such as Hungary, where abortion was accepted as a means of population control. Meanwhile, therapeutic studies would be conducted to provide the sort of doses to be used in contraceptive trials, and approval to carry these out was obtained from the CSM in 1969. These studies included ICI 46,474 (now also referred to by its brand name Nolvadex) for the treatment of anovulation or menorrhagia associated with high levels of endogenous estrogen (to be carried out at Aberdeen, Manchester and the Women's Hospital in Chelsea), and of breast carcinoma in 30 menopausal and post-menopausal women (at the Christie Hospital in Manchester).

The preliminary reports received from Dr. Klopper in Aberdeen and Drs. Murray and Osmond-Clarke in London helped to cast further light on the drug's mechanism of action, showing that that tamoxifen was capable of inducing ovulation at higher dose levels, while at lower doses it tended to have an anti-estrogenic effect^29^. As to the Christie breast cancer trial, although two of the women complained about hot flushes (which was taken as evidence of its anti-estrogen effect), no toxicity was observed and the drug appeared to be well tolerated, even at the highest dose of 10 mg by mouth.

In her unpublished history of tamoxifen, Dora Richardson wrote of the team's excitement as the first trial results arrived. She described the news of the birth of a child to a woman who had been infertile for 12 years and had failed to respond to treatment with clomiphene as a “boost to morale^30^.” She also described how the team were encouraged by the results of the breast cancer trial, even though these results were not received with universal enthusiasm at ICI: Walpole and his colleagues were told that they were supposed to be looking for a contraceptive pill, not an anti-cancer agent! At a Development meeting on 28th August 1970, sales estimates and quantities of bulk drug were set at 2 kg for initial stocks. Richardson concluded from these figures that the Development Department obviously envisaged treating only “dead people,” an indication of the hopelessness of the condition as it was viewed at that time (as well as lack of faith or ignorance on the part of the Development team)^31^. However, fortunately, on the basis of the positive clinical results, the CSM granted the company permission to prolong the trials as well as extend them to other centers. By the end of 1970, 60 patients had been admitted to the Christie breast cancer trial, and of the 40 women who had been on the trial for more than 10 weeks, all had shown measurable and marked tumor regression. Although these results were comparable to those achieved with the established synthetic hormone diethylstilboestrol, the clinicians carrying out the trial, Drs. Todd and Cole, reported how impressed they were with the absence of toxicity and the low incidence as well as trivial nature of any side-effects (Cole et al., 1971), especially compared with other agents used in cancer at the time, which were often either toxic, or—in the case of breast cancer—tended to have androgenic effects, and in some instances were so intolerable that patients had been withdrawn from treatment^32^.

In return, the trials provided clinical material for laboratory studies of tamoxifen. By then, the estrogen receptor had been isolated and identified by Gorski (Gorski et al., 1968), and Walpole and his team developed a receptor protein-binding assay method^33^. However, in a clinical setting, it was felt that a radio-immunoassay was more specific for measuring blood-estradiol levels in patients given tamoxifen^34^. The receptor-protein binding assay was therefore mainly used for experiments in laboratory animals, and showed tamoxifen to be a competitive inhibitor of estradiol binding to the uterine receptor protein in rabbits and in mice. Receptors sensitive to anti-estrogen were also found in various parts of rats' brains, including the hypothalamus and the pituitary. The results of the receptor-protein binding experiments in both these test systems suggested that, like other anti-estrogens, the action of tamoxifen was due to a high association constant but low effectiveness of the complex it formed with estrogen receptors (i.e., it was a partial agonist, with high affinity but low intrinsic activity)^35^. This was a pharmacological action with which ICI researchers had become familiar in their work on the beta-blockers (Quirke, 2006). It helped to cast further light on the physiological processes at a molecular level^36^, and made tamoxifen a particularly useful research tool for investigations of hormone-dependent tumors (Jordan et al., 1972).

Rendered confident by the clinical and laboratory studies carried out so far, Walpole's team began planning trials in contraception, and the Nolvadex Development Programme was drawn up^37^. This would play an important part in the drug's transformation from quasi-orphan to blockbuster drug (Quirke, 2012b).

### The Nolvadex Development Programme (1971)

The “Development Programme” was an organizational innovation which standardized and codified the R&D process at ICI. It marked the transition from the “Proving Trial” to the “Development Trial Stage^38^,” thus helping to bring together the “R” and the “D” in R&D^39^. ICI's first Development Programme had been written up in 1964 for the beta-blocker propranolol (Inderal)^40^. It followed a series of quarterly development reports^41^, and coincided with the hitherto separate Research and Development Departments coming together under the responsibility of a single Director, the Technical Director, as well as with the creation of the CSD in 1963. It therefore was a response to both internal and external factors and stimuli.

The Nolvadex Development Programme, which came 7 years after the Inderal Development Programme, included 16 rubrics, describing the work done up to June 1971 (the date of the start of the Programme), making an assessment of the drug's potential market, and plans for future work:

1. Clinical trials
2. Further laboratory work
3. Analysis
4. Sales formulation
5. Packaging
6. Process development and manufacturing of bulk drug
7. Manufacture and packing of tablets
8. Position in North America
9. Launch dates
10. Registration
11. Competitive situation
12. Sales estimates
13. Trade Mark and approved name
14. Patents
15. Publications
16. R&D costs

Three important considerations were taken into account when planning future work. First and foremost were tamoxifen's possible clinical uses, based on the results of trials received to date. These included: treatment of estrogen-dependent mammary carcinoma; induction of ovulation on women suffering from infertility due to failure to ovulate; menstrual disorders associated with abnormal levels of endogenous estrogen; oral contraceptive (a) for women, (b) for men; treatment for oligospermia; test for pituitary function; others. Secondly, the drug's position in North America was under question, following Ayerst's rejection of ICI's offer of Nolvadex for the American market, and the FDA's likely negative attitude toward its use in breast cancer. This attitude may have been due to a 1971 report in *JAMA* which had suggested that there was a link between diethylstilbestrol and a rare form of vaginal cancer, and was promptly followed by an FDA bulletin warning against the use of DES (FDA, 1971). Thirdly, the commercial situation, shown in Table 1, indicated that a number of therapeutic treatments of hormone-dependent breast cancers were already in existence, each of which commanded almost equal shares of the market.

**Table 1:** UK major branded products.

	**Annual sales (NHS level)**	**Cost of 1 week's treatment**
Provera 100 (Progestogen, Upjohn)	£50,000	£3.15
Masteril (Anabolic/androgen, Syntex)	£45,000	£1.35
Deca-durabolin (Anabolic/androgen, Organon)	£35,000	£0.75
Durabolin (Anabolic/androgen, Organon)	£28,000	£0.50
SH420 (Progestogen, Schering)	£27,000	£0.70

*Source: AZ PH 19597 B: Nolvadex Development Programme (June 1971)*.

Despite such competition from rival firms in America and Europe, tamoxifen had two advantages on which its market position would ultimately depend in relation to breast cancer: (1) its unique mode of action in being an estrogen-antagonist without androgenic properties, and since at the time it was the only product of its type its use should be larger; (2) it possessed very low incidence of side-effects compared with other forms of treatment. Another important consideration was that of past R&D costs (shown in Table 2), which had a bearing on budgeting and planning for future expenditure.

**Table 2:** Nolvadex R&D costs (£'000).

	**1964**	**1965**	**1966**	**1967**	**1968**	**1969**	**1970**	**1971**	**Est 1972**
Biology	6.7	1.7							
Toxicology	9.6	9.5	10.0	2.2	1.7			**1.1**	
Biochemistry	1.8	1.7	2.7		2.4	7.2	**8.3**	**8.0**	**2.0**
Analytical	1.8		0.2	0.9	0.4	0.2	**1.5**	**5.0**	**3.0**
Formulation							**0.4**	**4.8**	**5.0**
Process development	1.9			0.1			**5.0**	14.7^*^	
Medical				0.5	0.7	2.8	**3.5**	**5.6**	**8.0**
Development				0.5	0.4	0.5	**1.0**	**1.7**	**2.0**
Totals	21.8	12.9	12.9	4.2	5.6	10.7	**19.7**	**40.9**	**20.0**

* *Including cost of bulk drug*.

*Source: AZ PH 19597B: Nolvadex Development programme (June 1971)*.

The gaps in particular columns and rows in Table 2 exemplify the non-linear nature of pharmaceutical R&D, with bottle necks and feedback loops when advances in one area are held up by, and then develop in response to those in another. They also illustrate the pivotal part played by drug regulation in shaping the research and development activities of pharmaceutical firms. The trials that followed the CSM's approval for Nolvadex in 1969 not only led to an increase in existing expenditure in areas such as biochemistry, but to new expenditure in areas such as formulation (shown in bold).

As well as further trials in anovulatory infertility (in Abderdeen, Oxford, London, and Dublin), and in breast cancer (Manchester, Glasgow, and London), the Nolvadex Development Programme included plans for trials in contraception. “In view of the reluctance of British gynecologists” to become involved in such trials, in 1971 ICI contacted Professor Egon R. Diczfalusy, co-founder and Director of the WHO Research and Training Centre on Human Reproduction at the Karolinska Institute in Stockholm^42^, where he had already carried out collaborative projects involving healthy human volunteers using estrogens and other compounds^43^.

The Swedish trials led to the finding that, contrary to what might be expected from the laboratory studies in rats, tamoxifen stimulated rather than suppressed ovulation, and therefore would not work as a contraceptive pill in women. The market for a fertility drug was small, as seemed the market for an anti-cancer drug, partly due to the poor prognosis associated with the disease. Despite growing clinical evidence of the usefulness of tamoxifen in breast cancer, the very low sales estimates produced by the Marketing Department suggested that it was never going to cover the R&D costs and bring an appropriate return to the company. ICI's Main Board therefore made the decision to close down the Programme, but tamoxifen's champion, Walpole, threatened to resign. On this announcement, despondency spread through the entire research department. Moreover, when informed of the company's decision, one clinician said that, in view of the encouraging trial results, ICI could not *morally* withdraw the drug^44^. By then, the breast cancer trials had led to a number of publications, which sparked world-wide interest in tamoxifen^45^. Under such pressure, the company reversed its decision, Walpole remained, and the project was saved. In February 1973 ICI applied for a product license, which was granted a few months later, and in October of that year Nolvadex was launched in the UK for both anovulatory infertility and the palliative treatment of breast cancer. Although there continued to be crossovers between the two projects, the rest of this paper will focus on breast cancer. It will show how tamoxifen was transformed from a research object and palliative therapy for advanced breast cancer, into a diagnostic and predictive tool, an adjuvant chemo-endocrine treatment first in post-menopausal, then also in pre-menopausal women with early breast cancer, and eventually into the first chemopreventative for cancer.

### Tamoxifen, from palliative care to adjuvant therapy (1973–75)

Among the large number of clinical trials now being carried out with tamoxifen, Dr. Einhorn's studies at the Karolinska Institute in Stockholm had included a measurement of the rate of DNA synthesis in breast tumors and the effect this had on treatment. As a result, his group had been able to anticipate clinical response to, or relapse after, treatment with tamoxifen. From these observations, Walpole concluded that tamoxifen could be employed in pre-menopausal women with breast cancer for a short period as a tool to predict the usefulness of drastic treatments such as ophorectomy in these women. At the same time, he began making plans for a trial with Dr. J. C. Heuson of the European Breast Cancer Group, who was anxious to compare tamoxifen with Nafoxidine (an Upjohn compound which like tamoxifen could bind to the estrogen receptor, but unlike tamoxifen had several toxic side effects). The trial would include estrogen receptor determinations on biopsies taken from each patient to determine whether there was a correlation between clinical response to the compound and the presence of estrogen receptors in the tumor tissue^46^. By then, the clinical trials in fertility and contraception had also shown that in some instances tamoxifen led to the suppression of lactation. Walpole felt that this action would be of interest in the context of breast cancers which may be associated with high blood prolactin levels, and indeed at Westminster Hospital two patients who had responded well to tamoxifen had tumors which were thought to be prolactin-dependent^47^. Taken together, these observations on the measurement of DNA synthesis before and after treatment, of the content of estrogen receptors in breast tumors, and of blood prolactin levels led to the hope that it would be possible to predict the type of patient likely to respond to treatment with tamoxifen, i.e., to develop what is now referred to a “stratified therapy” (i.e., a re-branding of what was formerly known as “personalized medicine”; Smith, 2012).

However for this to happen, better screens had to be devised, first in animals and then in humans. In her unpublished history of tamoxifen, Dora Richardson commented that no laboratory tests for anti-tumor activity had been carried out with tamoxifen until *after* its activity in patients had been confirmed. The laboratory model adopted by Walpole's team to test for tumor inhibition was the DMBA (dimethyl benzanthracene) induced tumor in rats (also known as the Huggins tumor). The next step was to design a simplified method of receptor analysis, which could be applied routinely on a large scale in this model, before being applied in humans^48^. Walpole's team developed such a method in collaboration with Craig Jordan (from the Department of Pharmacology at Leeds University, who at the time was on leave of absence at the Worcester Foundation for Experimental Biology, USA, and whose work would later be sponsored by ICI; (Jordan, 2006), pp. 40–41). If it proved effective, i.e., if it demonstrated that tamoxifen could bind to the estrogen receptor in human breast tumors, it was hoped that this method would make it possible to screen patients for the presence of specific estrogen receptor in biopsy specimens of their tumors and to pre-select for treatment with Nolvadex those in whom such receptors had been found. However, alongside these highly scientific methods, clinicians continued to use observations such as “hot flushes” as indications that the treatment was working and remission was likely to occur^49^. Walpole therefore proposed that physiological indicators might also be used to ensure that individual patients were not being “under-treated” and could be given the maximum effective dose to produce an improved response^50^.

In his report of February 1974, Walpole wrote: “By good fortune, Nolvadex was launched at a time of increased interest in the assessment of the endocrine status in breast cancer^51^.” Tamoxifen was shown to be highly effective in binding to the estrogen receptor and, before long, researchers in Europe and the US were therefore using tamoxifen as a tool to “predict the response of breast tumors to hormone therapy^52^.” However, this new use for tamoxifen brought out the fact that not all patients whose tumors had demonstrable estrogen receptor levels responded well to endocrine therapy. Although this paradox might be due to the fact that the receptor assays used were not of consistent standard, it suggested that a number of biochemical events were a pre-requisite for complete endocrine regulation, and that other lesions occurred in patients for whom endocrine therapy failed, thereby casting further light upon the complex processes involved in malignant disease.

Nolvadex was also launched at a time when the value of chemotherapy in cancer was being established with novel drugs tested first alone, then combined, in collaborative multi-center trials (see Keating and Cambrosio, 2007; also Quirke, 2014, pp. 670–671). With drug resistance becoming a growing concern, not only in bacteria, but also in cancer cells, combination therapy was being developed and its modalities refined. Hence, in June 1974, Walpole began planning a trial in which two different treatment modalities, supposedly devoid of cross-resistance, would be used, and he proposed to alternate their administration on a 4-week basis^53^. The rationale for this trial was that, unlike conventional sequential treatments, each alternating treatment would be started *before* rather than *after* the effect of the previous one was exhausted, thus resulting in a cumulative effect. Two added benefits of such an approach were that (1) drugs with high levels of toxicity, such as adriamycin and vincristin, could be given for much longer, and (2) at precise moments in the treatment cycle, the patient's bone marrow and immune system would have a chance to recover. This approach was tried by Dr. Heuson under the aegis of the European Organization for Research and Treatment of Cancer (EORTC, which had been created in 1962), alongside another trial in pre-menopausal women^54^.

Such plans and discussions, which were based on a growing number of publications and symposia presenting evidence not only of symptom relief, but also of remissions and survival from breast cancer^55^, indicate that, both as a research tool and a therapeutic agent, tamoxifen was shifting from palliative care into the realm of chemotherapy, transforming it in the process. What follows will concentrate on the years 1975–1980, after which ICI's research reports on tamoxifen and related topics ended. During that period Walpole was mainly involved in the Nolvadex Development Programme until his sudden death in 1977. Although his involvement ensured continuity between the research and development phases, Walpole's gradual disengagement from the research, which can be detected in the reports, meant that the project lacked clear purpose and direction. Months were lost to pressures of competing work inside the company, and aspects of the research were outsourced to external laboratories (Jordan, 2006, Chapter 3). Nevertheless, in that time, the foundations were laid for the next phase in tamoxifen's trajectory, from adjuvant therapy to the first chemopreventative remedy for cancer.

### 1975–1980: The final years of ICI's tamoxifen project

Clinical trials carried out in Britain by Ward (Birmingham) and Brewin (Glasgow) and beyond (in Germany) showed that the response to tamoxifen in patients who experienced a recurrence of their breast tumor after primary surgery and/or radiotherapy tended to increase with age^56^. These findings prompted the question of what the mechanism for this action might be, since tamoxifen was an “anti-estrogen.” Could it be that tamoxifen exerted an estrogenic action (albeit a weak one) by way of its metabolites?^57^ The study was taken up at ICI by Barry Furr^58^ and B. Valaccia, and a programme of synthesis and tests of analogs of tamoxifen metabolites in a number of different screens, not only estrogen, but also progesterone and androgen receptor screens, was initiated to find out whether tamoxifen could bind with them, and therefore be useful in other cancers. Later prostaglandin synthetase (PGS) inhibitor screens were also developed by the team. These showed that tamoxifen was an effective inhibitor of human breast tumor PGS in addition to arresting tumor growth, thus offering an explanation for the clinical observation that patients taking Nolvadex for advanced breast cancer often experienced relief from bone pain, and strengthening the rationale for its use in adjuvant chemotherapy further still. Hence, it was hoped as a result of this programme that a follow-up compound for Nolvadex might be found—the target being an anti-estrogen of similar potency to Nolvadex with one or more of the following properties in addition: lower agonist activity, shorter half-life, greater inhibitory activity against PGS, anti-androgenic activity^59^.

This new research strand, which would lead ICI to its second major breakthrough in cancer therapy: ICI 118,630 (goserelin, Zoladex), was stimulated by the discovery by Schering Plough of the first non-steroidal anti-androgen Flutamide for the treatment of prostate cancer. As they had done earlier with Merrell's drug, ICI therefore mobilized their synthetic capabilities and the scientific expertise acquired with tamoxifen to search for a non-steroidal anti-progestin (which unlike anti-androgens would have the advantage of having neither anti-anabolic activity nor any effects on “normal sexual behavior”)^60^. Another approach consisted in looking for a novel, potent analog of the luteinizing hormone-releasing hormone (LHRH), also referred to as the gonadotrophin-releasing hormone (GnRH), although this was initially expected to be used mainly in animal breeding^61^. As well as testing the compounds in the company's by now well established receptor-binding assays, once again the team needed to develop new *in vivo* screens, and “in view of the previous experience with Nolvadex, that is anti-estrogenic in the rat and estrogenic in mice,” tests would have to be carried out in more than one species. Because the chick comb was known to be androgen sensitive and chicks were cheap, it was chosen as one of the animal models in which to test active compounds and compare them to Flutamide.

Meanwhile, a special organization had been created for the purpose large-scale clinical studies of tamoxifen as an adjuvant treatment for cancer: the Nolvadex Adjuvant Trial Organisation (NATO). Until then, adjuvant therapy had consisted either in chemotherapy using mainly cytotoxic drugs, or in major endocrine ablation after curative surgery. Clinical trials of tamoxifen in adjuvant therapy therefore began in 1976, some progressing ahead of schedule, and their favorable results, which showed that Nolvadex was effective in both pre- and post-menopausal women regardless of their receptor status, were frequently discussed at symposia and in the medical press from 1977 onwards^62^. Not only did these results change the modalities of adjuvant therapy for breast cancer whilst helping to establish tamoxifen in the treatment of the early stages of the disease (NATO, 1983, 1988), but in the context of these adjuvant trials evidence also emerged of the drug's potential to *prevent* the recurrence of breast cancer in women at high risk (i.e., who had already had cancer in one breast). This potential was explored in a trial carried out in Denmark, with the aim of establishing the value of tamoxifen as a “prophylactic” in breast cancer (Andersen et al., 1981; Mouridsen et al., 1988). Patients were selected who had had a mastectomy with or without radiation and in whom there was no evidence of metastases, for it was known that 55–60% of them would develop local recurrence of the disease or metastases within 5 years. They were then randomly allocated either to Nolvadex, stilboestrol, or a placebo^63^. The trial eventually showed that although 10% of the women treated with placebo developed a recurrence of their breast cancer, none of those treated with tamoxifen had experienced such a recurrence^64^. Such results would later help to justify the initiation of breast cancer prevention trials, for instance the Breast Cancer Prevention Trial NSABP-P1 (BCPT), with the aim of establishing whether 5 years of tamoxifen would reduce the incidence of invasive breast cancer in women identified as being at high risk of the disease, and yet healthy (Fosket, 2010; Löwy, 2012; also Fosket, 2004).

Almost as soon as it had moved into the realm of cancer chemotherapy, tamoxifen therefore hinted at the theoretical and practical possibilities of chemoprevention in cancer. Further trials would turn tamoxifen into the first preventative for any cancer, helping to establish the broader principles of chemoprevention, while extending the market for tamoxifen and similar drugs further still (Early Breast Cancer Trialists Collaborative Group, 1992, 1998)^65^.

### Tamoxifen, from the clinic into the medical marketplace

Thanks to tamoxifen, ICI were able to tap into the vast cancer research network connected in Europe through the EORTC, and across the Atlantic through the National Cancer Institute (NCI). The interest tamoxifen generated among scientists and clinicians, rather than the promotional activities of the company, which Dora Richardson argued remained very limited, greatly enhanced its position in the medical marketplace. In a personal communication to Walpole, Dr. Scott Lippman of the NCI had described his method for testing tamoxifen in human breast cancer cell lines which were dependent on estrogens for their long-term growth in tissue culture^66^. In these cells, tamoxifen showed itself to be strongly inhibitory of both DNA and protein synthesis. Lippman had turned this method into a “kit” for measuring receptors, and spurred by their American subsidiary (ICI-USA), ICI did not waste time in starting work on their own quantitative assay “kit to be marketed as an adjunct to Nolvadex.” Such a kit would not only make money for itself, but by helping to justify the use of tamoxifen, would further enhance the market position for the drug, particularly in the USA^67^. By then, the company had submitted an Investigational New Drug (IND) application to the Food and Drug Administration (FDA). It was followed in 1976 by a New Drug Application (NDA) to the FDA's Oncological Drugs Advisory Committee, in which John Patterson, a member of ICI's Clinical Research Department (formerly of the Medical Department), made a detailed and convincing case for Nolvadex in breast cancer^68^. By 1984, the NCI were describing tamoxifen as the adjuvant chemotherapy of choice for breast cancer (Consensus Conference, 1985). Although ICI's application for a US patent for tamoxifen had originally been rejected on the basis that the US Patent Office did not recognize advances on existing inventions, and that Merrell's patent for clomiphene pre-dated that for tamoxifen, in 1985 the American court of appeals finally granted ICI the patent rights for tamoxifen in the USA, thereby starting the 17-year patent cover there, paradoxically at a time when it was coming to an end in other countries (Jordan, 2006, p. 40).

Tamoxifen's entry into the American market contributed to rising worldwide sales: although ICI's Marketing Department had only expected it to make £100,000 p.a. in 1970, by 1974 figures on the home market alone amounted to £140,000, overtaking one of ICI's well established drugs Mysoline (for epilepsy). By 1976, sales figures were equivalent to those for the anesthetic Fluothane, the first drug to put ICI's Pharmaceutical Division “in the black,” and for over-the-counter drugs such as, the antiseptic Savlon. As the expiry date for their tamoxifen patents was drawing near, in 1979 ICI obtained a 4-year extension for their UK patent, on the basis of “the nature and merits of the invention in relation to the public,” as well as “the profits made by the patentee^69^.” By 1980, it was making £30 M for the firm^70^.

Nevertheless, even as late as September 1982, at the annual portfolio review attended by the managers of the Biology Department (Dr. J. D. Fitzgerald) and Chemistry (Dr. R. Clarckson), the manager of the Marketing Department, who also attended the meeting, commented that “there was no market for cancer^71^.” ICI's Marketing Department were not alone in under-estimating the market for anti-cancer drugs: if tamoxifen had not been “stolen” by American companies while it remained unprotected by patents, it was partly because they did not believe in its usefulness either (Jordan, 2006, p. 40). The fate of tamoxifen therefore rested on the qualities of the drug itself, and the interest it generated not only among researchers both inside and outside the company, but also among patients and the wider public. As mentioned earlier in this paper, Dora Richardson's history of Nolvadex was—quite unusually for such an internal publication—accompanied by letters from patients who attributed their lives to tamoxifen. Appendix 6 entitled “What do the patients think” included a letter to ICI's Pharmaceutical Division, in which a grateful patient wrote: “Thank you for a miracle.”

Tamoxifen benefited not only from being the first of a kind, which helped to confer upon it the status of a “miracle drug,” but once again from its origins as a contraceptive pill. As the name indicates, it could be taken orally, and this mode of administration meant that Nolvadex was suitable for home treatment, and a large proportion of sales (75%) occurred through retail pharmacies. This enabled local tinkering with established protocols, as well as a degree of self-experimentation, as testified by another letter, written by a cancer researcher (Dr. June Marchant of the Regional Cancer Registry, West Midlands Oncology Group), who had been diagnosed with breast cancer, and having spent 20 years in cancer research was well versed in the modalities of cancer therapy^72^. After discussing her ideas with her clinician, whom she described as “understanding,” together they worked out “an unconventional management programme.” Because her thymus gland was within the radiation field of her breast tumor she refused radiation therapy. Instead, she decided to undergo therapy with a new cytotoxic drug that was being tested locally in a clinical trial. She appeared to make an uneventful recovery, but in 1972 a scan revealed metastases in her brain. At this point, she therefore elected local treatment with radiation of the head and adjuvant therapy with tamoxifen. Knowing from her own research that prolactin had been identified as a hormone with perhaps an even greater significance than estrogen in the maintenance of breast tissue and breast tumor growth, she started reading the relevant literature. A number of inhibitory substances had been tried on a few patients with breast cancer, and among them Levodopa appeared to give beneficial results. Her clinician therefore agreed to give her Levodopa as additional anti-hormonal therapy. Her drug regimen was phased out in 1975, and at the time of writing her letter, in 1976, the author felt “very well indeed, having had no ablative operation, cytotoxic drugs or masculinizing hormones.” From her own experience, she therefore concluded that “systemic therapy, in addition to local therapy, had a vital role to plan in the management of the disease,” and she wished to share this positive experience with others.

Her conclusions went beyond ascertaining the value of tamoxifen in adjuvant therapy—extrapolating from her experience with the drug, she defended “systemic therapy” more generally. Yet, after Dr. Stephen Carter, who had been responsible for ICI's cancer project on Cell Division and Growth^73^, left the company, taking early retirement in 1979, he was not replaced, and the project on cell growth was terminated. Thus, in 1980, when tamoxifen was bringing in sizeable profits for the company and Zoladex (for prostate cancer) was in the pipeline, ICI had no longer a cancer research programme, a situation that lasted until in 2006, when Alderley Park became the Global Lead Centre for the company's cancer research^74^.

## Discussion

If tamoxifen made it into the medical marketplace, it was largely *despite* rather than because of the company's marketing department. Thanks to having inside a drug champion prepared to risk his career to save his project and a medical department willing to run the gauntlet of the FDA to promote tamoxifen in the USA, but also thanks to interest generated outside, among scientists, clinicians, and patients who asked for or agreed to take the drug, it was transformed from a failed contraceptive pill into a successful breast cancer medicine. The patients' letters referred to in this essay provide us with a unique insight into this transformation, but also into the public demand and experimentation which escape the control of both the industry and the professions, and are not normally included in discussions of pharmaceutical innovation.

Focusing on the early history of tamoxifen has made it possible to examine in some detail both the brakes and the stimuli for pharmaceutical innovation. These come from inside as well as outside industry, contrary to a rather narrow model of pharmaceutical innovation, according to which companies, motivated by a commercial more than a scientific agenda, push drugs onto an unsuspecting public, often with the connivance of the medical profession, but hopefully kept in check by the actions of regulatory authorities (for example see: Crawford, 1988; Marsa, 1997; Law, 2006).

In the case of tamoxifen, pharmaceutical innovation was predominantly science- and clinic-driven, rather than market-driven (so a case of demand-pull rather than supply-push, Walsh, 1994). It benefited from a number of coincidences: its ability to bind to the newly-discovered estrogen receptor helped to make it into a useful tool for investigating hormone-dependent tumors, as well as a drug of choice for treating breast cancer. It was developed at a time when palliative care was becoming an important part of cancer treatment (Clark, 2007), and when chemotherapy was successfully being applied to cancer in collaborative trials. These placed ICI at the center of a global network of cancer institutions and organizations, which helped to maintain interest in their drug even as ICI was losing its research focus on cancer. Hence the last phase in tamoxifen's transformation, into the first chemopreventative for cancer, owed more to this global network than to ICI's efforts at promoting their drug. Finally, tamoxifen was developed at a time when cancer patients were encouraged to demand better treatments, to become more proactive in their own care, and engage with ideas of risk.

In the beginning, when tamoxifen was being developed as a contraceptive pill, cancer patients had to some extent been used as “proxies” for normal, healthy human subjects, and their voices were mostly heard trough the clinicians who reported on their symptoms as indications of the drug's activity and side-effects. Nevertheless, the fact that their voices were included, both indirectly in the reports and directly in Dora Richardson's history of Nolvadex, suggests that to the company these voices did matter: they helped to shape the content of the research, whilst justifying it, both morally and scientifically. Rather than a “detour” in relation to contraception (Oudshoorn, 2002), the study of tamoxifen in breast cancer was therefore carried out in close parallel with its study in contraception (and subsequently fertility). This is not surprising, given that ICI's interest in cancer pre-dated their interest in contraception by 20 years. Nevertheless, the contraception project helped to determine tamoxifen's fate as a drug: from what it was (a synthetic anti-estrogen, safe with a relatively low incidence of side-effects), to how it could be taken (orally, and therefore suitable for home treatment).

Thus, the drug and its fate were shaped by the industrial setting from which it emerged. In return, tamoxifen transformed the biomedical landscape in which it was deployed. As it moved from contraception into cancer, tamoxifen expanded its market at the same time as its clinical role, transforming cancer therapy in the process. Not only did it cast fresh light on the function of sex hormones and their role in malignant disease, but it hinted at the possibility of personalized medicine, and helped to lay the foundations of chemoprevention. Indeed, although the concept of chemoprevention had already begun to take hold with drugs to treat cardiovascular diseases (to lower cholesterol or blood pressure, for instance), by becoming associated with and tapping into the drive to catch cancer early by screening and, even better, prevent it by introducing life-style and other changes, the principles and practice of chemoprevention were further strengthened by drugs like tamoxifen and their application to the field of cancer.

In the context of cancer chemoprevention, the question of its use in normal, healthy women arose once more, but it did not go unchallenged. In her chapter on “Breast Cancer Risk as Disease”, Jennifer Fosket has described the controversies that surrounded the BCPT which took place in the USA in the 1990s, highlighting the fact that the risks associated with tamoxifen were often downplayed, and this despite letters from ICI (which had spun off its pharmaceutical division to form Zeneca) warning both doctors and women enrolled on the trials that—by then—some women taking tamoxifen had developed endometrial cancer (Fosket, 2010, p. 345). Although the BCPT identified an increased risk of pulmonary embolism, deep-vein thrombosis, as well as endometrial cancer in women who had taken tamoxifen compared to the control group on placebos, their findings were nonetheless favorable to tamoxifen: only 124 women had developed breast cancer in the tamoxifen group, compared to 244 in the placebo group (Fisher et al., 1998). On the other hand, the results of the Royal Marsden Study, carried out in the UK at roughly the same time as the BCPT, were not so clear-cut: they revealed no significant reduction in breast cancer incidence in women at risk who took tamoxifen (Powles et al., 1998). These different results were attributed to key differences between the American and European trials, ranging from their organization, to the numbers of women enrolled, the criteria for their selection, and different conceptualizations of what constituted “high risk^75^.” Such differences and controversies surrounding the trials led the FDA to downgrade its approval from “prevention” to “reduction of risk” (Fosket, 2010, p. 348).

In a sense then, tamoxifen had been the victim of its own success. Originally intended for women with little chance of survival, its ability to cause disease in women experiencing long-term remissions thanks to tamoxifen led to a complex assessment of risk, which had to be shared with women undergoing treatment for breast cancer. Thus, in 1996, a guide written for clinicians and patients on the subject of tamoxifen stressed the importance of communicating the risks involved in taking the drug, from minor side effects such as hot flushes, to potentially serious ones including other cancers. Hence, what was nevertheless a message of hope did not only relate to tamoxifen itself, but also to the “new patient” which caring professionals, breast cancer advocates, and the media had helped to create: “prepared with background information about the disease”; requiring “treatment options”; wanting “good communication and information” and wanting “the truth” (Langer, 2006, p. 134). Such patients did exist, as we saw in the case of June Marchant, even though she may have been exceptional, and in many ways drugs like tamoxifen had also helped to bring them about.

## Concluding remarks

The focus of this paper on the industrial context for the development of tamoxifen highlights the importance of the early phases in the history of pharmaceutical innovation, for this early history shapes the form and content of drugs, has the potential to define their use and ultimately determine their fate in the medical marketplace, and this despite the many twists and turns that characterize their trajectory from bench to bedside.

This particular focus also throws into sharp relief the contribution made by applied research to the advancement of scientific knowledge: in the case of tamoxifen, more specifically to the understanding of basic physiological processes involved in human reproduction and malignant disease. Such a contribution is in part due to the fact that industry, perhaps more easily than academia with its rigid disciplinary boundaries, enables a to-ing and fro-ing between separate, yet contiguous research projects and therapeutic areas (in this instance, between contraception, fertility, and cancer). This to-ing and fro-ing between projects illustrates once again the non-linear nature of pharmaceutical innovation. Typified by blind alleys, fresh departures, feedback loops between the laboratory and the clinic, as well as serendipitous discoveries, the early history of tamoxifen brings to the fore the role of human agency, the institutional memory that is often associated with long-term investment in particular areas of expertise, and is embodied in individual researchers like Walpole.

Just as the industrial context is worthy of historical enquiry, the early history of drugs such as, tamoxifen, which are at once emblematic and idiosyncratic examples of pharmaceutical innovation, may yield useful lessons for potential innovators, by helping them to identify key moments when choices are made and decisions taken, so that these may in time be revisited and alternative paths may be explored. For innovators are at once the makers and the products of history, even if history is often remote from their concerns or absent from their writings. Unfortunately, because of the growing difficulty of accessing pharmaceutical archives, this rich vein of historical enquiry may fast be coming to an end. The hope remains that, as an essential component of their intellectual capital, such archives will continue to be available to researchers both inside and outside companies.

## Topic editors' declaration

This article is classified as “Original Research” as it reports on primary sources of a historical nature, including previously unpublished studies.

### Conflict of interest statement

The author declares that the research was conducted in the absence of any commercial or financial relationships that could be construed as a potential conflict of interest.

## Footnotes

^1^In 2003 it was estimated that 400,000 women were alive thanks to the drug, and millions have benefited from extended disease-free survival (See Jordan, 2003).

^2^AstraZeneca, formerly ICI (hereafter AZ), research and other reports: Oral Contraception (AZ CPR 70: 1960-64); Endocrinology and Fertility (AZ CPR 101: 1965-72); Viruses and Cancer (AZ CPR 54-55: 1958-64). These unpublished reports were made accessible to me between 2002 and 2009 by kind permission from AstraZeneca.

^3^D. N. Richardson, “The history of Nolvadex” (AZ PH27039 B, 13 May 1980).

^4^Richardson, “The history of Nolvadex.”

^5^There compounds were later understood to work by alkylation—i.e., the transfer of an alkyl group from one molecule to another, in the case of anti-cancer agents attaching it to DNA, thus inhibiting cancer cell division—hence such compounds became known as “alkylating agents” because of their mechanism of action.

^6^Since the 1930s it was known that viruses can induce tumors in laboratory mice. The oncogenic potential of a number of virus groups, including adenoviruses, herpesviruses, and poxviruses, was identified in the 1950s and 1960s (See Rigby and Wilkie, 1985).

^7^Walpole, “The technical possibility of oral contraception” (AZ ICP 10695, 13 June 1956).

^8^G.D. Searle's norethynodrel, brand name Enovid, had been patented in 1955, and Syntex's norethisterone, brand name Ortho-Novum, was patented in 1956. NB: both were progesterone analogues containing some oestrogen (See Marks, 2001, pp. 72–73).

^9^Walpole, “Steroids as oral contraceptives” (PD/B 353, 13 Nov. 1958); AZ CPR 70 “Oral Contraception.”

^10^Richardson, “The history of Nolvadex”—MER 25 was also later shown in clinical trials to have low potency and unacceptable side effects.

^11^A. L. Walpole and G.E. Paget, “Pituitary inhibitors” (AZ CPR 70/1B: Oral Contraception Sep. 1960).

^12^Walpole et al. “Pituitary Inhibitors” (AZ CPR 70/2B: Endocrinology, June 1961). NB: the reports were re-named “endocrinology” after the study of steroids and their action on cholesterol metabolism was included in the project.

^13^Richardson, “The history of Nolvadex.”

^14^Cholesterol levels had become a serious concern since Merrell's new drug MER 29 (Triparanol) had been found to cause irreversible cataracts by interfering with cholesterol biosynthesis, and had had to be withdrawn from the market. B. W. Langley and A. L. Walpole (AZ CPR 70/5B: Endocrinology May 1963). See also Richardson, “The history of Nolvadex.”

^15^B. W. Langley and A. L. Walpole (AZ CPR 70/5B: Endocrinology May 1963).

^16^The compounds were covered by patents GB 1013907 and 1064629.

^17^Quoted in Jordan (2006), p. 39.

^18^Walpole et al. “Reproduction” (AZ CPR 70/6B: Endocrinology, June 1964).

^19^John Patterson, personal communication, 20 April 2009.

^20^Richardson, “The history of Nolvadex.” It was later discovered that this effect disappeared by the time of weaning (Jordan, 2006).

^21^Walpole et al. “Reproduction” (AZ CPR 70/6B: Endocrinology June 1964).

^22^After it had been found to be estrogenic in sheep, in 1970 the compound was even considered as a fattening agent for livestock. Walpole et al. (AZ CPR 101/16B Endocrinology and Fertility 20 Feb. 1970).

^23^AZ CPR 101/1B Endocrinology and Fertility January 1965.

^24^Ibid.

^25^Walpole et al. (AZ CPR 101/20b Endocrinology and Fertility 28 June 1971). These changes were measured by radio-immunoassay (plasma estradiol) as well as protein binding (progesterone) once appropriate tests had been developed.

^26^AZ CPR 101/7B Endocrinology and Fertility, January 1967.

^27^Its introduction is said to have begun the era of assisted reproductive technology. See Dickley and Holtkamp (1996).

^28^It was thought that British gynecologists would be unlikely to come across more than one woman a year to whom abortion could be offered under the new law. Walpole et al. (AZ CPR 101/19B Endocrinology and Fertility, 19 Feb. 1971).

^29^Walpole et al. (AZ CPR 101/17B Endocrinology and Fertility 25 June 1970).

^30^Richardson, “The History of Nolvadex.”

^31^Ibid.

^32^Walpole et al. (AZ CPR 101/18B Endocrinology and Fertility, 28 Oct. 1970). Norethisterone was a drug with such an androgenic effects. See Richardson, “The history of Nolvadex.”

^33^Walpole et al. (AZ CPR 101/17B Endocrinology and Fertility 25 June 1970).

^34^Walpole et al. (AZ CPR 101/19B Endocrinology and Fertility 19 Feb. 1971).

^35^Tamoxifen is now classed as a selective estrogen receptor modulator (SERM), meaning that it activates estrogen receptors in some tissues, while blocking them in others.

^36^Walpole et al. (AZ CPR 101/18B Endocrinology and Fertility 28 Oct. 1970); idem, (AZ CPR 101/19B Endocrinology and Fertility 19 Feb. 1971).

^37^AZ PH 19597 B: Nolvadex Development Programme, June 1971.

^38^“Minutes of a meeting of the ICI 46,474 Development Team, Development Department, held on 28th August 1970” (Appendix 4 of Richardson, “The history of Nolvadex”).

^39^AZ PH 19597 B: Nolvadex Development Programme, June 1971.

^40^AZ PH15355B: Inderal Development Programme, 15 June 1964.

^41^AZ CPR 68B: Research and Development Quarterly Reports (1962-1964).

^42^Ian Askew, “Obituary: HPR co-founder, Egon R. Diczfalusy, 1920–2016,” http://www.who.int/reproductivehealth/obituary-e-diczfalusy/en/ (accessed 02.06.17).

^43^Walpole et al. (AZ CPR 101/20B, 28 June 1971). On the WHO Research Centre see: http://ki.se/en/kbh/who-center-for-human-reproduction (accessed 02.06.17). See also Oudshoorn (1998).

^44^Richardson, “The History of Nolvadex”; see also Jordan (2003).

^45^Walpole et al. (AZ CPR 101/26B Fertility, 22 June 1973).

^46^Ibid.

^47^The presence of prolactin in human blood had been confirmed by Henry Friesen et al. (1970).

^48^Walpole et al. (AZ CPR 101/27B Fertility 24 Oct. 1973).

^49^Walpole (AZ CPR 101/28B Fertility 26 Feb. 1974).

^50^Walpole (AZ CPR 101/28B Fertility 16 June 1974).

^51^This interest was stimulated by observations such as Jense's (Jense et al., 1971), who showed that patients with tumors containing high affinity estrogen receptors were more likely to have a remission following adrenalectomy than those without such receptors (with remissions in 10/13 patients with positive tumors, but in only 1/26 patients without).

^52^Walpole et al. (AZ CPR 101/32B Fertility 27 June 1975). One of these researchers was W. L. McGuire in the USA. See McGuire et al. (1975).

^53^Walpole (AZ CPR 101/28B Fertility 14 June 1974).

^54^Walpole (AZ CPR 101/32B Fertility 27 June 1975). For more on the history of EORTC see: http://www.eortc.org/history/ (accessed 05.06.17). Its journal, the *European Journal of Cancer*, was launched in 1965.

^55^Bonadonna et al. “Cytotoxic chemotherapy for mammary cancer,” Symposium, Padova, 8 Apr. 1974, quoted in Walpole (AZ CPR 101/28B Fertility 16 June 1974).

^56^Walpole (AZ CPR 101/32B Fertility 27 June 1975).

^57^Gregory (AZ CPR 101/34B Fertility 17 Feb. 1976).

^58^“Professor Barry Furr” Obituary: https://alumni.reading.ac.uk/our-alumni/dr-barry-furr (accessed 10.06.17).

^59^Crossley (AZ CPR 101/37B Fertility 27 Jan. 1977).

^60^Ibid.

^61^Ibid.

^62^Richardson, “The history of Nolvadex.”

^63^Walpole (AZ CPR 101/32B Fertility 27 June 1975).

^64^Richardson “The history of Nolvadex”.

^65^NB: the FDA approved tamoxifen for the reduction of breast cancer risk in 1998.

^66^Walpole, (AZ CPR 101/32B Fertility 27 June 1975).

^67^Crossley (AZ CPR 101/33B Fertility 2 Oct. 1975). Such kits, which would now be referred to as “companion diagnostic tests,” remain a mainstay of breast cancer therapy today.

^68^Richardson, “The History of Nolvadex.”

^69^UK 1949 Patents Act, Section 23. http://www.legislation.gov.uk/ukpga/1949/87/pdfs/ukpga_19490087_en.pdf (accessed 15.06.17). I thank Dr. Michael Jewess for pointing out this section to me.

^70^Richardson, “The History of Nolvadex.”

^71^Dr. J.D. Fitzgerald, personal communication.

^72^Dr. June Marchant, “Personal view” (27 November 1975), Appendix 6 of Richardson, “The History of Nolvadex.”

^73^AZ CPR 110 (1965-77): Cell Division and Growth.

^74^On Alderley Park, see AstraZeneca, “Alderley Park, Cheshire,” http://www.pharmaceutical-technology.com/projects/astrazeneca-alderley/ (accessed on 15.06.17). This has become Manchester's new bioscience campus: https://mspl.co.uk/campuses/alderley-park/ (accessed on 15.06.17), while AZ's UK R&D center has been relocated to Cambridge see: https://www.astrazeneca.com/our-science/cambridge.html (accessed 15.06.17).

^75^Foskett has suggested that Royal Marsden selected women based on their family history, and this may have led to more women with the BRCA1 and 2 gene mutations, for whom tamoxifen is a less effective preventative, being enrolled in the trial (Fosket, 2010, n. 2 p. 352).
